# Myocardial blood flow and viability in children post palliation of hypoplastic left heart syndrome assessed with MRI

**DOI:** 10.1186/1532-429X-17-S1-O58

**Published:** 2015-02-03

**Authors:** Philip Wegner, Michael Jerosch-Herold, Christopher Hart, Eileen Pardun, Hans-Heiner Kramer, Carsten Rickers

**Affiliations:** 1 Department of Congenital Heart Disease and Pediatric Cardiology, Schleswig-Holstein University Hospital (UK-SH), Campus Kiel, Kiel, Germany; 2 Radiology, Brigham and Women - Harvard Medical School, Boston, MA, USA; 3 Pediatric Cardiology, Asklepios, St. Augustin, Germany

## Background

Myocardial blood flow in the systemic right ventricle in Hypoplastic Left Heart Syndrome (HLHS) is largely unknown. We examined regional and global myocardial perfusion reserve, viability and function in HLHS after completion of Fontan circulation using MRI.

## Methods

In 42 HLHS patients (6.0 ±2.3 yrs) and 14 healthy volunteers (6.8 ±3.9 yrs), MRI first-pass perfusion (0.03 mmol/kg Gd-DTPA; TR/TE/α=2.6/1.1/20°) and late gadolinium enhancement (LGE) imaging was performed using a 3 Tesla scanner (Philips Achieva). Quantitative myocardial blood flow at rest and stress (Adenosin 140 mcg/kg/min) was calculated in 4 anatomical RV segments per slice using a model independent deconvolution. A total of 672 segments were analysed. CMR results were compared to conventional x-ray guided coronary angiography in all HLHS pts.

## Results

HLHS patients showed impaired myocardial perfusion reserve (MPR, hyperemic/resting flow) as compared to the right or left ventricle of healthy children (2,4 ±0,6 vs. 3,1±0,9 (RV); p<0,05 or 2,58±0,70 vs. 3,4±1,13 (LV); p<0,01). HLHS subgroups with a large rudimentary LV (n=25) showed lower septal perfusion, areas of non-viable myocardium (20.8 vs 3%) and a lower cardiac index (2.3±0.7 vs. 3.3±0.8 l/m2/min; p<0,05). The diameter of the pre-coronary segment of the hypoplastic aorta did not correlate with myocardial blood flow. All HLHS patients had patent epicardial coronary arteries.

## Conclusions

The global impairment of coronary flow reserve in HLHS patients after Fontan may indicate altered vasoreactivity due to extensive aortic surgery. Furthermore, in HLHS subgroups with a large rudimentary LV, scar tissue and a reduced cardiac index can be observed. These findings may be of prognostic significance for the long-term outcome in HLHS.

## Funding

N/A.

